# Bio-Inspired Adhesive Hydrogels for Localized Therapeutic Delivery: From Catechol Chemistry to Smart Biointerfaces

**DOI:** 10.3390/biomimetics11080593

**Published:** 2026-08-20

**Authors:** Hee Sook Hwang, Chung-Sung Lee

**Affiliations:** 1 Department of Pharmaceutical Engineering, Dankook University, Cheonan 31116, Republic of Korea; 2 Department of Pharmaceutical Engineering, Soonchunhyang University, Asan 31538, Republic of Korea; 3 Institute for Molecular Metabolism Innovation, Soonchunhyang University, Asan 31538, Republic of Korea

**Keywords:** bio-inspired adhesive hydrogel, catechol chemistry, localized therapeutic delivery, smart biointerfaces, regenerative medicine

## Abstract

Localized therapeutic delivery has gained increasing attention as an effective strategy in enhancing treatment efficacy while at the same time minimizing systemic side effects. However, conventional hydrogel-based therapeutic delivery systems often suffer from poor tissue retention and insufficient control over delivery. This drawback is highly pronounced in wet and dynamic biological environments. So, catechol-based adhesive hydrogels have emerged as promising biomaterials for localized therapeutic applications and are inspired by the remarkable wet-adhesion capability of marine mussels. As a highlight, catechol chemistry enables robust tissue adhesion through multiple intermolecular interactions, including hydrogen bonding, metal coordination, and covalent coupling. At the same time, it contributes to hydrogel cohesion and structural stability. Recent advances in hydrogel engineering have expanded the functionality of these systems through integration of injectable formulations, self-healing networks, nanocomposite reinforcement, and stimuli-responsive biointerfaces. These developments have transformed adhesive hydrogels from tissue sealants into multifunctional therapeutic platforms capable of enhancing tissue retention, regulating therapeutic release, and dynamically interacting with biological microenvironments. Here, we review molecular mechanisms underlying catechol-mediated adhesion and discuss recent progress in advanced adhesive hydrogel systems. We further highlight their therapeutic applications in wound healing, musculoskeletal regeneration, exosome and gene delivery, immunomodulatory therapies, and localized cancer therapy. Finally, current translational challenges and future opportunities in developing next-generation smart biointerfaces for precision regenerative medicine are discussed.

## 1. Introduction

Localized therapeutic delivery has emerged as attractive strategy in improving treatment efficacy and simultaneously minimizing systemic adverse effects in wide range of diseases and tissue disorders. This delivery enables sustained retention of therapeutic agents at target sites, enhances local bioavailability, and reduces off-target exposure compared to conventional systemic administration [1,2]. These advantages are particularly important in regenerative medicine, wound healing, musculoskeletal repair, and localized cancer therapy. In all these cases, prolonged interactions between therapeutic cargos and damaged tissues are often required to achieve optimal clinical outcomes [3,4]. However, maintaining therapeutic agents at the desired site remains a significant challenge due to rapid diffusion, biological clearance, and the dynamic nature of physiological environments [5,6]. These challenges often outweigh the substantial advances in drug delivery technologies.

So, hydrogel-based therapeutic delivery systems have been extensively proposed as biomimetic matrices capable of providing localized and sustained therapeutic release and have been investigated to address the above-mentioned limitations [5,7]. Hydrogels possess unique characteristics, including high water content, tunable physicochemical properties, and structural similarities to native extracellular matrix (ECM). These qualities make them highly suitable for biomedical applications [5,8]. Nevertheless, conventional hydrogels frequently exhibit insufficient tissue adhesion. This is, however, a serious problem, leading to premature delivery agent detachment, inadequate retention, and reduced therapeutic efficacy, particularly in wet and mechanically active tissues [9,10].

As a solution, bio-inspired adhesive hydrogels have emerged to overcome these challenges and are inspired by natural biological adhesion systems, as their name implies. In particular, mussel-inspired catechol chemistry has attracted considerable attention among various bioadhesive strategies [11,12]. For example, adhesive proteins secreted by marine mussels contain abundant 3,4-dihydroxy-L-phenylalanine (DOPA) residues, whose catechol groups can participate in multiple intermolecular interactions. These interactions include hydrogen bonding [13], metal coordination, π–π stacking, and covalent crosslinking [9,13]. In this context, these versatile interactions enable strong and durable adhesion to diverse biological substrates, while simultaneously contributing to hydrogel cohesion and mechanical integrity.

Recent advances in material engineering have transformed adhesive hydrogels into multifunctional therapeutic platforms beyond the scope of simple tissue adhesion [12,14]. Notably, catechol-functionalized polymers have been combined with dynamic crosslinking networks, inorganic nanomaterials, nanoparticles, extracellular vesicles, and bioactive therapeutics to create advanced hydrogel systems. The hydrogels are, in particular, created with enhanced mechanical performance, controllable degradation, and programmable therapeutic release [5,15]. Furthermore, integration of stimuli-responsive mechanisms has enabled the development of smart biointerfaces capable of responding to local physiological signals and modulating therapeutic functions in a spatiotemporally controlled manner [16,17]. These emerging design strategies are expanding the potential of adhesive hydrogels from conventional wound sealants to next-generation platforms for precision regenerative medicine and localized therapy.

We review recent advances in bio-inspired adhesive hydrogels for localized therapeutic delivery here. We place particular emphasis on catechol-mediated adhesive mechanisms and hydrogel engineering strategies. We first summarize the molecular basis of catechol-driven adhesion and its application in hydrogel network design. We then highlight emerging developments in injectable, self-healing, nanocomposite, and stimuli-responsive adhesive hydrogel systems. Finally, we discuss representative therapeutic applications and future opportunities for development of smart biointerfaces that enable more effective and personalized approaches to localized therapy and tissue regeneration.

## 2. Catechol-Based Adhesive Mechanisms

### 2.1. Molecular Basis of Catechol-Mediated Adhesion

**Figure 1:**
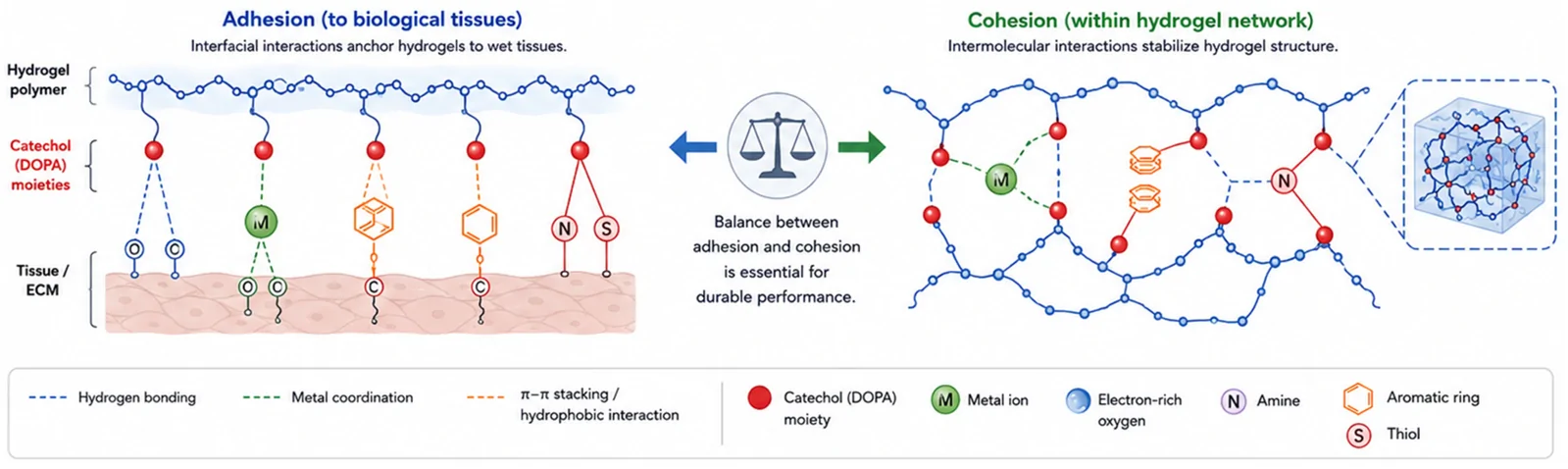
Dual roles of catechol chemistry in hydrogel adhesion and cohesion. Catechol moieties promote hydrogel adhesion to biological tissues through hydrogen bonding, metal coordination, π–π stacking/hydrophobic interactions, and quinone-mediated covalent coupling. The same interactions within hydrogel networks function as reversible or permanent crosslinking mechanisms. Such mechanisms usually provide structural integrity, self-healing capability, and mechanical robustness. The multifunctional chemistry of catechol enables multiple simultaneous interactions in aqueous environments and contributes synergistically to both adhesion and cohesion.

The exceptional adhesive capability of marine mussels has provided a powerful blueprint for the development of bio-inspired adhesive materials. Meanwhile, conventional synthetic adhesives often exhibit limited performance in hydrated environments. On the contrary, mussels achieve robust and durable attachment to a wide variety of organic and inorganic substrates even under continuous exposure to seawater [12,13]. This unique wet-adhesion phenomenon pertaining to mussels is primarily attributed to mussel adhesive proteins (MAPs), which contain high concentrations of 3,4-dihydroxy-L-phenylalanine (DOPA) [12,18]. Notably, the catechol functionality of DOPA has been identified as a key molecular determinant responsible for versatile and adaptive adhesion behavior prevalent in mussel plaques (Figure 1 and Table 1) [9,11,18].

A distinguishing feature of catechol is its chemical promiscuity, which enables a single functional group in it to participate in multiple intermolecular interactions simultaneously. Usually, catechol groups readily establish hydrogen bonds with hydroxyl, carboxyl, phosphate, and amine functionalities on biological surfaces and at hydrated tissue interfaces [3,10]. These interactions facilitate intimate interfacial contact and contribute to initial adsorption of catechol-containing materials. More importantly, catechol possesses an extraordinary ability to displace interfacial water molecules. This ability helps overcome one of the major barriers to wet adhesion by simply promoting direct molecular contact between adhesive materials and tissue surfaces. The result is substantially enhanced interfacial binding strength [19,20].

In addition to hydrogen bonding, catechol exhibits strong affinity toward metal ions through reversible coordination interactions [12]. The formation of mono-, bis-, and tris-catechol–metal complexes with ions such as Fe^3+^, Ti^4+^, Ca^2+^, and Mg^2+^ contributes to substrate adhesion and dynamic reinforcement of hydrogel networks [21,22]. Metal-catechol coordination bonds are particularly attractive in biomedical hydrogel design because they provide reversible crosslinking, self-healing capability, and energy dissipation under mechanical stress [22,23,24].

The adhesive versatility of catechol is further enhanced by its redox activity [12]. In detail, catechol can be converted into highly reactive quinone species under oxidative conditions. Catechol oxidation can proceed gradually through ambient oxygen or be accelerated by enzyme-mediated pathways involving oxidases such as tyrosinase and laccase [25,26]. The oxidation pathway and kinetics can influence the extent of quinone formation and subsequent crosslinking, whereas excessive or rapid oxidation may reduce available catechol groups and potentially compromise cytocompatibility [27]. These species can then establish covalent coupling with nucleophilic groups, such as amines, thiols, and imidazoles, found in extracellular matrix proteins and cell membrane components [3,9,28]. The corresponding reactions here generate stable covalent linkages at the tissue-material interface, significantly improving long-term retention and adhesive durability. Simultaneously, catechol oxidation can induce intermolecular catechol-catechol coupling, leading to cohesive network formation within hydrogel matrices. The dynamic interplay between reduced catechol and oxidized quinone states therefore governs both adhesive and structural properties of bio-inspired hydrogels.

Generally, effective bioadhesion requires simultaneous optimization of interfacial adhesion and bulk cohesion from a materials engineering perspective [12,29]. It is known that insufficient internal cohesion can result in premature mechanical failure and loss of functionality, even though strong interfacial interactions are preferred to ensure attachment to biological tissues. Conversely, excessive crosslinking may compromise conformal contact and reduce adhesive performance [29,30]. Catechol chemistry offers a unique molecular platform capable of balancing these competing requirements. Such balancing is achieved through integration of reversible non-covalent interactions, dynamic coordination bonding, and covalent crosslinking mechanisms within a single chemical motif of catechol [9,30].

Overall, the multifunctional nature of catechol-mediated interactions provides a molecular foundation for the design of advanced bio-inspired adhesive hydrogels. Robust wet adhesion, dynamic network stabilization, and tunable interfacial properties are all available in catechol chemistry in general. These aspects and qualities make it a central strategy for developing localized therapeutic delivery systems and next-generation smart biointerfaces capable of operating within complex biological environments.

### 2.2. Catechol-Functionalized Hydrogel Networks

**Figure 2:**
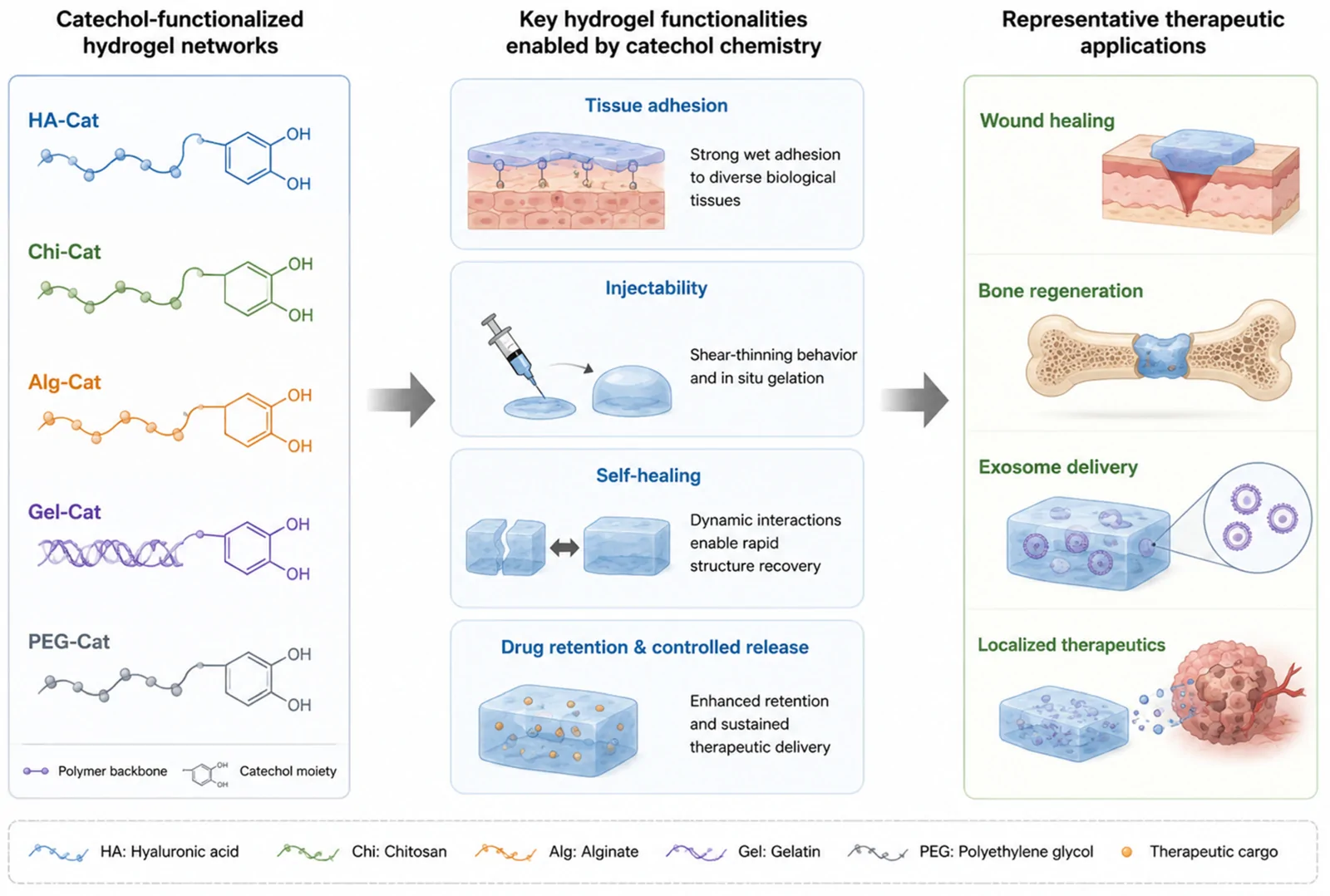
Design framework of catechol-functionalized hydrogel networks for localized therapeutic delivery. Diverse natural and synthetic polymer backbones can be functionalized with catechol groups to generate bio-inspired adhesive hydrogels. These hydrogels exhibit key functionalities including robust tissue adhesion, injectability, self-healing, and controlled therapeutic retention through catechol-mediated interfacial interactions and dynamic network formation. Synergistic integration of polymer-specific biological functions and catechol-driven adhesion enables a broad spectrum of biomedical applications ranging from tissue regeneration to advanced localized therapeutic delivery.

Incorporation of catechol moieties into polymeric backbones has become one of the most widely adopted approaches for engineering bio-inspired adhesive hydrogels [10,12]. Specifically, catechol performs the primary adhesive function, and the physicochemical and biological characteristics of the resulting hydrogel are derived from the parent polymer. Therefore, selection of an appropriate polymeric backbone plays a critical role in mechanical properties, degradation behavior, cellular interactions, and therapeutic performance of the hydrogel (Figure 2) [3,10,31]. So far, a variety of natural and synthetic polymers have been functionalized with catechol groups in the past decade to generate adhesive hydrogel systems tailored to localized drug delivery, tissue regeneration, and biomedical interfacing (Table 2) [3,29].

Hyaluronic acid-catechol (HA-Cat) systems have attracted considerable attention compared to other materials mentioned in Table 2 owing to the intrinsic biological functions of hyaluronic acid (HA) [32]. Notably, HA exhibits favorable biocompatibility, biodegradability, and hydration capacity and interacts with cell-surface receptors such as CD44 as a major component of the extracellular matrix [33,34]. Functionalization with catechol groups significantly enhances the adhesive properties of HA without compromising its biological advantages. Consequently, HA-Cat hydrogels have been extensively studied for wound healing, cartilage repair, bone regeneration, and localized therapeutic delivery [33,35,36]. Furthermore, the abundance of reactive functional groups within HA enables incorporation of various crosslinking strategies, nanoparticles, growth factors, extracellular vesicles, and therapeutic agents for the hydrogel. Eventually, this incorporation facilitates development of multifunctional hydrogel platforms with enhanced tissue retention and regeneration [36].

Natural polysaccharides such as chitosan and alginate have also been widely modified with catechol groups to improve their adhesive performance [37,38,39]. Chitosan-catechol (Chi-Cat) hydrogels combine the intrinsic mucoadhesive, antimicrobial, and hemostatic properties of chitosan with the wet-adhesion capability of catechol [38,40,41]. These characteristics make Chi-Cat particularly attractive for wound management, mucosal drug delivery, and tissue sealing. Similarly, alginate-catechol (Alg-Cat) hydrogels leverage the favorable gelation behavior and biocompatibility of alginate and overcome its inherently weak tissue adhesion. This is another specific case supporting the fact that catechol modification can improve interfacial interactions with biological tissues to enhance retention at target sites and enable more effective localized therapeutic delivery [41,42].

Protein-derived biomaterials have also been extensively considered as adhesive hydrogel matrices [43,44,45,46]. Gelatin-catechol (Gel-Cat) systems are particularly attractive because gelatin retains numerous bioactive motifs derived from collagen. One such motif is the arginine-glycine-aspartic acid (RGD) sequence that promotes cell adhesion and tissue integration. Specifically, the combination of intrinsic bioactivity and catechol-mediated adhesion enables Gel-Cat hydrogels to establish stable tissue interfaces and support cellular infiltration and matrix remodeling. As a result, these systems have been widely investigated within tissue engineering, wound healing, and regenerative medicine where both bioactivity and adhesive performance are required [42,47,48].

In addition to naturally derived polymers, synthetic materials such as polyethylene glycol (PEG) have been functionalized with catechol groups to generate highly controllable adhesive hydrogels [49,50]. PEG offers excellent batch-to-batch reproducibility, tunable molecular architecture, and precisely adjustable mechanical properties, unlike natural polymers. Catechol-functionalized PEG hydrogels provide robust tissue adhesion and maintain design flexibility associated with synthetic polymer networks. These characteristics have facilitated the development of injectable hydrogels, surgical sealants, and controlled drug delivery platforms with predictable physicochemical properties and reproducibility [51,52,53,54,55].

Integration of catechol moieties into diverse polymeric backbones has enabled the development of adhesive systems with highly tunable biological and physicochemical characteristics. Notably, catechol acts as a versatile molecular tool complementing intrinsic properties of each polymer rather than serving solely as an adhesive functional group. However, the choice of polymer backbone introduces distinct design trade-offs. Natural polymers generally provide favorable biocompatibility and biological functionality but may exhibit compositional variability and limited mechanical tunability, whereas synthetic polymers offer greater control over network architecture and mechanical properties but often lack intrinsic bioactivity. Therefore, selection of the polymer backbone should be guided not only by adhesive performance but also by the mechanical, biological, and degradation requirements of the intended therapeutic application.

### 2.3. Factors Governing Adhesive Performance

Overall adhesive performance of catechol-functionalized hydrogels is influenced by multiple physicochemical and structural parameters of their constituents, even though the major molecular foundation for the performance is provided by catechol chemistry. Adhesive strength, tissue retention, mechanical stability, and therapeutic functionality of these systems are determined by catechol groups and also by the dynamic interplay between network architecture, crosslinking mechanisms, and environmental conditions within the systems [12,29]. Therefore, understanding the key factors governing the adhesive behavior is essential for rational design of hydrogel platforms tailored to localized therapeutics.

One of the most critical parameters of the above-mentioned behavior is catechol content within the polymer network. Increasing the degree of catechol functionalization generally enhances the availability of adhesive sites, leading to stronger interactions with biological tissues and improved wet adhesion [3,31]. However, excessive catechol incorporation may accelerate intermolecular aggregation and oxidative crosslinking, potentially reducing chain mobility and effective interfacial contact [3,49].

The oxidative state of catechol also plays a pivotal role in determining the adhesive performance in the concerned hydrogels. Notably, reduced catechol groups primarily contribute to reversible interfacial interactions such as hydrogen bonding and metal coordination. However, oxidation of catechol generates reactive quinone species capable of forming covalent linkages with nucleophilic groups in tissues and proteins [3,10,28]. Consequently, the catechol-to-quinone ratio significantly influences both short-term adhesion and long-term retention. Environmental factors including pH, oxygen availability, reactive oxygen species, and enzymatic activity can further modulate catechol oxidation kinetics and ultimately affect the adhesive behavior [28,56].

Crosslinking density is another important design parameter governing both adhesion and cohesion. Increasing crosslinking density generally improves mechanical integrity and structural stability. However, excessive network formation may restrict polymer chain mobility and reduce the ability of the hydrogel to establish intimate contact with irregular tissue surfaces [5,29]. Conversely, insufficient crosslinking can compromise cohesive strength, resulting in premature failure under physiological stress. Therefore, achieving an appropriate balance between network stability and interfacial adaptability is crucial for improved adhesive performance.

Mechanical properties of hydrogels used in tissues are closely associated with the effectiveness of tissue adhesion. Hydrogels intended for such biomedical applications must hence possess sufficient mechanical robustness to withstand physiological forces and maintain conformal contact with surrounding tissues. In particular, viscoelastic behavior, energy dissipation capacity, and self-healing characteristics of the hydrogels have emerged as important determinants of their long-term adhesive functionality [17,21,57]. Dynamic interactions such as catechol-metal coordination and reversible covalent bonding are frequently exploited to enhance mechanical resilience without sacrificing adhesive capability in these hydrogels.

Degradation behavior of adhesive hydrogels further influences their durability and therapeutic utility. Excessively rapid degradation may result in insufficient therapeutic retention, and overly stable networks may hinder tissue remodeling and regeneration [8,58,59]. Therefore, degradation kinetics should be carefully tailored according to intended clinical application. This is in line with the understanding in regenerative medicine that controlled degradation is particularly important for synchronizing material persistence with tissue healing and sustained therapeutic delivery.

Taken together, adhesive performance in these hydrogels is governed by a complex interplay among catechol content, oxidation state, polymer network architecture, mechanical characteristics, and related degradation behavior (Table 3). Rational optimization of these parameters is essential for developing bio-inspired adhesive hydrogels that simultaneously achieve robust tissue adhesion, structural stability, and effective localized therapeutic delivery. Representative quantitative parameters reported for catechol-functionalized adhesive hydrogel systems are further compared in Table 4, although direct comparison should be interpreted cautiously because experimental conditions and characterization methods vary across studies.

## 3. Emerging Designs of Bio-Inspired Adhesive Hydrogels

**Figure 3:**
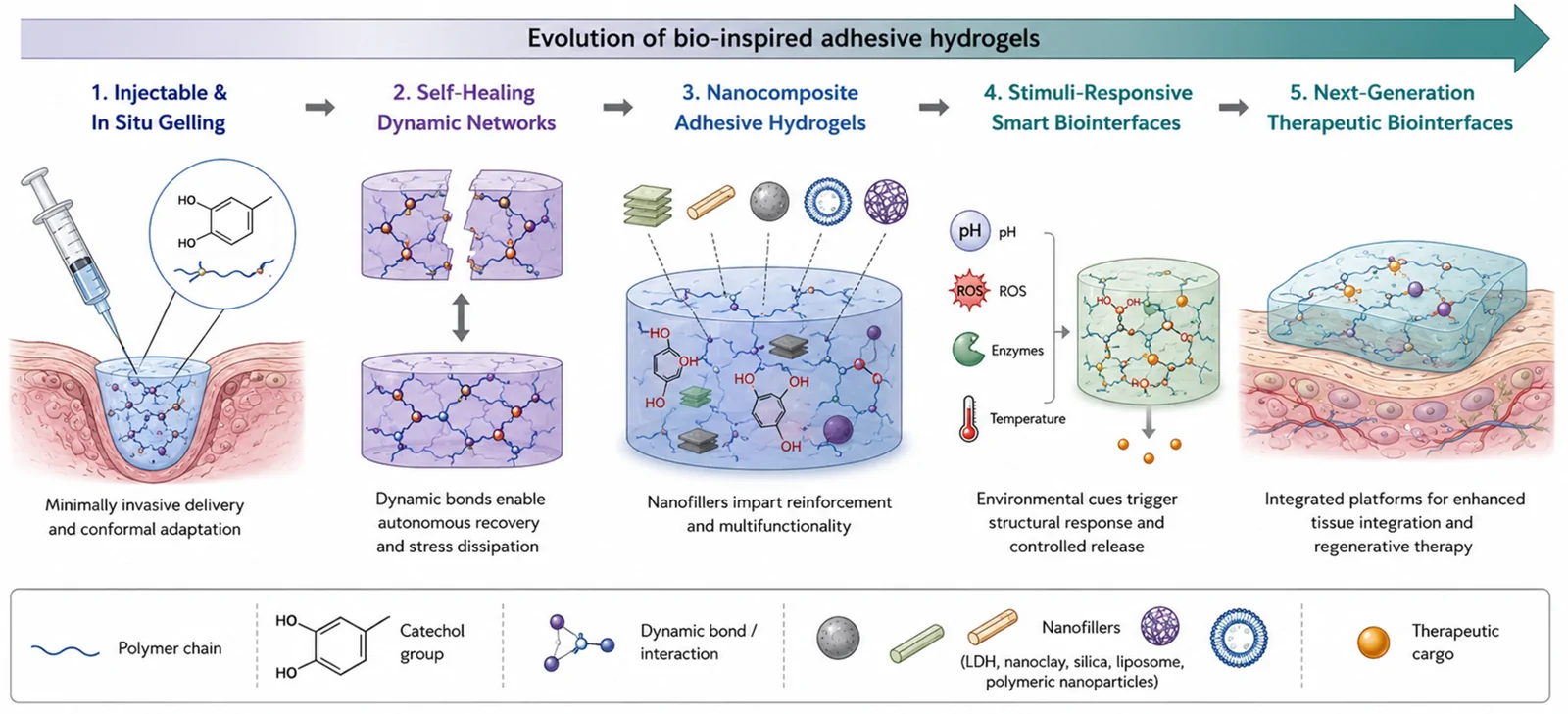
Evolution of bio-inspired adhesive hydrogels to smart therapeutic biointerfaces. Advances in hydrogel engineering have progressively transformed catechol-based adhesive hydrogels from simple tissue-adhesive materials to multifunctional therapeutic platforms. Integration of injectability, dynamic self-healing networks, nanocomposite reinforcement, and stimuli-responsiveness enables enhanced tissue retention, mechanical adaptability, and controlled therapeutic release. These emerging design strategies support the development of next-generation smart biointerfaces for localized therapeutic delivery and regenerative medicine.

Building on catechol-mediated adhesion, recent hydrogel engineering has focused on integrating injectability, dynamic self-healing, nanocomposite reinforcement, and stimuli-responsive functions to address the mechanical and therapeutic limitations of conventional adhesive systems [5,57,73,74,75]. These emerging design strategies have hence transformed conventional adhesive hydrogel ideas into multifunctional therapeutic platforms capable of improving tissue retention, mechanical adaptability, and spatiotemporal control of therapeutic release [17,73,74,75]. These strategies enable improved tissue retention, mechanical adaptability, and spatiotemporal control of therapeutic delivery, as summarized in Figure 3.

### 3.1. Injectable and Self-Healing Hydrogels

Clinical translation of adhesive hydrogels requires more than strong tissue adhesion alone. So, hydrogel systems must also be capable of minimally invasive administration, conformal adaptation to irregular tissue geometries, and long-term mechanical stability under physiological conditions as part of effectively functioning within complex biological environments. These requirements have driven the development of injectable and self-healing hydrogel systems that combine bio-inspired adhesion and dynamic network architectures [5,17]. Integrating injectability, in situ gelation, and autonomous network recovery makes these advanced hydrogels emerge as promising platforms for localized therapeutic delivery and regenerative medicine.

Injectable hydrogels offer significant advantages over preformed implants and conventional surgical materials [76,77]. Their ability to be administered through syringes or catheters enables minimally invasive delivery and ensures homogeneous distribution within complex defect sites [78,79,80]. Injectable systems can fill irregular tissue cavities and establish intimate contact with surrounding biological structures following their administration. These characteristics hence maximize tissue retention and therapeutic localization. In a real case scenario of this, Zhou et al. developed a horseradish peroxidase (HRP)/hydrogen peroxide (H_2_O_2_)-crosslinked, injectable, catechol-modified chitosan hydrogel. This hydrogel in particular was made to be delivered through a syringe, rapidly gel in situ, conform to irregular cartilage defects, and promote stable tissue integration and cartilage regeneration in vivo [38]. Catechol-functionalized hydrogels are particularly attractive in this context because catechol-mediated interfacial interactions promote rapid tissue adhesion immediately after injection. This promotion is instrumental in reducing material displacement and improving local therapeutic efficacy [49,55].

A key feature of many injectable adhesive hydrogels is their capacity for in situ gelation [81]. Moreover, these hydrogels undergo sol-to-gel transition directly at the target site in response to chemical, physical, or biological triggers, unlike pre-crosslinked materials. Various gelation mechanisms have been explored for this context, including catechol oxidation, Schiff-base formation, enzymatic crosslinking, and metal-catechol coordination [82,83,84,85]. Also, in situ gelation not only improves retention at the administration site but also facilitates encapsulation of sensitive therapeutic cargos such as proteins, extracellular vesicles, and nucleic acids. Representative examples of these hydrogels include mussel-inspired injectable adhesive hydrogels based on alginate–dopamine/chondroitin sulfate/regenerated silk fibroin networks. This hydrogel undergoes rapid enzymatic in situ gelation and enables sustained exosome encapsulation and release for the promotion of endogenous cell recruitment and cartilage regeneration [82].

Emergence of dynamic covalent networks has further expanded the functionality of adhesive hydrogels [57]. Dynamic bonds such as Schiff-base linkages, boronate ester interactions, and reversible metal-catechol coordination provide adaptable network structures capable of continuously reorganizing according to physiological conditions [86]. Among these strategies, dynamic Schiff-base chemistry based on reversible reactions between aldehyde and primary amine groups is particularly attractive for constructing injectable and self-healing adhesive hydrogels [87,88,89]. The reversible formation and exchange of imine bonds facilitate shear-thinning injection and network recovery after mechanical disruption, while their combination with dopamine- or catechol-functionalized components can provide wet tissue adhesion through complementary interfacial interactions [54,87,88,89]. Moreover, the dynamic and pH-sensitive nature of imine bonds can enable pH-responsive network changes and controlled therapeutic release under acidic conditions [90,91]. In one case of this material, Guo et al. developed a catechol-functionalized polysaccharide hydrogel based on multiple dynamic interactions, including Schiff-base bonding, borate–diol interactions, and hydrogen bonding. The developed hydrogel, in particular, was fortified with rapid self-healing, shear-thinning injectability, shape adaptability, and durable tissue adhesion for infected wound healing [89]. Also, these dynamic networks can dissipate mechanical stress, accommodate tissue movement, and maintain structural integrity over extended periods, unlike pre-crosslinked materials. Such characteristics are particularly important in mechanically active tissues where repetitive deformation can compromise the performance of conventional hydrogel systems.

As a next step, self-healing hydrogels have attracted growing attention as next-generation adhesive biomaterials built upon the advancements discussed so far [92]. Self-healing of these hydrogels enables them to autonomously recover their structural and functional properties after any mechanical damage, without external intervention [93,94]. This capability is typically achieved through reversible molecular interactions, including dynamic covalent bonds, supramolecular interactions, and metal-ligand coordination. In this context, Zhong et al. developed a catechol-grafted chitosan hydrogel crosslinked through dynamic borate ester bonds and hydrogen bonding. This hydrogel in particular exhibited rapid self-healing within seconds, excellent mechanical recovery, and robust tissue adhesion. These properties are a demonstration of how reversible dynamic interactions can substantially improve durability and reliability of adhesive hydrogels [95]. In addition to enhancing durability, self-healing properties contribute to prolonged therapeutic retention and improved reliability of localized therapeutic delivery systems [96]. These advantages are particularly relevant in regenerative medicine, where biomaterials are often exposed to prolonged mechanical stress and dynamic biological remodeling.

So, integration of injectability, in situ gelation, dynamic crosslinking, and self-healing represents a major evolution in the design of bio-inspired adhesive hydrogels (Table 5). These interconnected design strategies enable hydrogels to achieve effective tissue adhesion while simultaneously addressing practical challenges associated with delivery, retention, and long-term functionality. As a result, injectable and self-healing hydrogels have become foundational platforms for the development of advanced localized therapeutics and smart regenerative biomaterials. However, these properties are not necessarily optimized simultaneously: rapid gelation may improve post-injection retention but restrict defect conformability, whereas highly dynamic networks favor injectability and self-healing at the potential expense of long-term mechanical stability. Thus, the relative contribution of permanent and reversible crosslinks should be tailored to the mechanical and therapeutic requirements of the target tissue.

### 3.2. Nanocomposite Adhesive Hydrogels

Practical application of catechol-functionalized hydrogels is often limited by their insufficient mechanical strength, rapid degradation, and limited control over therapeutic release despite their excellent adhesive properties [49]. Therefore, increasing attention has been given to the incorporation of nano-scale building blocks into adhesive hydrogel matrices as a means of addressing the above-mentioned challenges. The resulting nanocomposite adhesive hydrogels from relevant efforts in this regard combine the tissue-adhesive characteristics of catechol with the structural and functional advantages of nanomaterials. So, this combination enables the development of multifunctional platforms with enhanced mechanical performance, therapeutic retention, and biological activity.

Layered double hydroxides (LDHs) have emerged as particularly attractive compounds out of several available ones in adhesive hydrogel engineering [97,98,99]. Notably, their high surface area, tunable composition, and ion-exchange capability enable LDH nanosheets to interact with polymer chains through multiple physicochemical mechanisms. Eventually, this interaction reinforces hydrogel networks and improves structural stability. LDHs can function as therapeutic reservoirs and network modulators that regulate swelling behavior, degradation kinetics, and cargo diffusion, apart from being mechanical reinforcements [79,97]. Representative studies on LDHs have demonstrated that integrating bioactive LDH nanoparticles into hydrogel matrices enhances the mechanical stability of the hydrogel network and provides sustained release of therapeutic cargos. It also promotes osteogenic differentiation and angiogenesis. For example, gelatin methacryloyl (GelMA)–LDH nanocomposite scaffolds loaded with calcein significantly improved bone regeneration through synergistic effects of structural reinforcement and bioactive ion-mediated therapeutic delivery [100]. Such multifunctional roles distinguish LDHs from conventional reinforcing fillers and highlight their potential in developing advanced localized therapeutic delivery systems.

Nanoclay-based hydrogels represent another important class of nanocomposite adhesive materials [101]. Exfoliated silicate nanosheets can establish extensive physical interactions with polymer chains, resulting in improved toughness, shear-thinning, and self-healing [98,102]. Furthermore, nanoclay incorporation can enhance catechol-mediated cohesive interactions and improve hydrogel stability under different physiological conditions [103]. In this case, Guan et al. developed a Laponite-reinforced catechol-functionalized chitosan hydrogel in which nanoclay nanosheets cooperatively strengthened the dynamic polymer network, enhanced wet tissue adhesion, and imparted injectable and self-healing properties [104]. The resulting nanocomposite hydrogel further promoted angiogenesis and accelerated infected wound healing. These capabilities demonstrated the multifunctional contribution of nanoclay beyond mechanical reinforcement. These findings have led to widespread investigation of nanoclay-containing adhesive hydrogels for use in tissue engineering, wound healing, and injectable biomaterials.

Silica nanoparticles have also been extensively explored in the fabrication of mechanically reinforced adhesive hydrogels [105,106]. Surface-functionalized silica nanoparticles provide abundant interaction sites for polymer networks and can improve the hydrogel compressive strength, viscoelasticity, and structural durability [107]. In addition, their tunable surface chemistry allows their integration with catechol-mediated adhesive mechanisms. This integration then facilitates the design of multifunctional hydrogel systems with improved tissue retention and therapeutic performance. For example, Zheng et al. incorporated nanoscaled bioactive glass (nBG), a silica-based nanoparticle, into a catechol-modified thermo-sensitive hydrogel. In this material, the nanoparticles not only reinforced the hydrogel network but also promoted angiogenesis and wound healing while at the same time preserving excellent injectability and tissue adhesion. This study hence illustrates how silica-based nanomaterials can simultaneously improve structural and biological performances of adhesive hydrogels [108].

More recently, soft nanomaterials such as liposomes and polymeric nanoparticles have been incorporated into adhesive hydrogels to simultaneously achieve structural support and therapeutic functionality [109,110]. These nano-scale carriers serve as localized drug depots capable of protecting sensitive cargos and regulating release kinetics, unlike inorganic nanomaterials that primarily enhance mechanical properties [111]. Integration of nanoparticle-mediated delivery with adhesive hydrogel matrices then creates hierarchical therapeutic systems that combine prolonged tissue retention with sustained and localized therapeutic release. Representative studies under this class of materials have further demonstrated that integrating stimuli-responsive liposomes into adhesive hydrogel matrices can help achieve spatiotemporally controlled therapeutic delivery. For example, Wang et al. developed a blood-triggered catechol-based adhesive hydrogel incorporating reactive oxygen species (ROS)-responsive liposomes. In this material, the hydrogel rapidly achieved hemostasis and tissue adhesion, and the embedded liposomes released therapeutic cargo on demand under oxidative stress [112]. This hierarchical design effectively combined prolonged tissue retention and environment-responsive drug release, highlighting the therapeutic potential of soft nanocarrier-integrated adhesive hydrogels.

In essence, the evolution of nanocomposite adhesive hydrogels reflects a shift from passive reinforcement to multifunctional network design. Contemporary nanomaterials increasingly serve as active structural and biological regulators that influence hydrogel mechanics, degradation behavior, therapeutic transport, and cell-material interactions. Such synergistic integration of catechol-mediated adhesion and nanomaterial-enabled functionality has rendered nanocomposite hydrogels a key platform for next-generation localized therapeutic delivery and regenerative medicine.

### 3.3. Stimuli-Responsive Smart Biointerfaces

Recent advances in bio-inspired adhesive hydrogels have extended their functionality beyond passive tissue adhesion and therapeutic retention to dynamic interactions with biological microenvironments [49]. Stimuli-responsive hydrogels are inspired by the adaptive behavior of living tissues. Hence, they are capable of sensing local physiological or pathological cues and responding through changes in their network structure, mechanical properties, or therapeutic release profiles [113,114]. Such adaptive behavior has led to the emergence of smart biointerfaces that actively communicate with surrounding tissues and regulate therapeutics in a spatiotemporally controlled manner.

pH-responsive hydrogels have attracted considerable interest among various differently strategized stimuli-responsive hydrogels. Reliance of these hydrogels on pH is very beneficial, as there are several abnormal pH conditions associated with many pathological environments, including chronic wounds, inflammatory tissues, and tumors [5,57]. Changes in local pH can, in general, alter polymer ionization, crosslinking density, and network swelling, thereby triggering release of encapsulated therapeutics [16]. For example, Shan et al. developed a phenylborate–catechol crosslinked adhesive hydrogel in which reversible dynamic covalent bonding enabled pH-dependent gel degradation and controlled drug release. The gel also simultaneously maintained self-healing capability and tissue adhesion [115]. In essence, this study highlights how pH-responsive catechol chemistry can couple environmental sensing and programmable therapeutic delivery. pH-dependent oxidation of catechol groups in catechol-functionalized hydrogel systems can further influence adhesive interactions and network stability, providing additional opportunities for environmentally responsive therapeutic regulation.

ROS-responsive hydrogels represent another rapidly growing area of research [16,116]. Elevated ROS levels are characteristic of numerous pathological conditions, including chronic inflammation, tissue injury, and degenerative diseases [117]. Incorporation of ROS-sensitive linkages in these hydrogels enables hydrogel degradation or network remodeling in response to oxidative stress. These results then facilitate site-specific therapeutic release while simultaneously scavenging excessive ROS [118]. In a real scenario, Zhou et al. developed a dual glucose/ROS-sensitive catechol-based injectable adhesive hydrogel crosslinked through dynamic phenylborate bonds [119]. ROS-triggered cleavage of phenylborate linkages under oxidative conditions in these hydrogels promoted on-demand release of therapeutic nanoparticles while simultaneously scavenging excessive ROS. Hence, these operations reduce inflammation and enhance diabetic wound healing. This particular study hence illustrates how ROS-responsive network engineering can integrate environmental sensing with adaptive therapeutic regulation in bio-inspired adhesive hydrogels. Such dual functionality is particularly attractive in regenerative medicine where modulation of oxidative microenvironment can directly influence tissue repair.

Enzyme-responsive hydrogels exploit disease-associated enzymatic activities to achieve selective therapeutic activation [120,121]. Notably, matrix metalloproteinases (MMPs), hyaluronidases, and other tissue-remodeling enzymes are frequently overexpressed during wound healing, inflammation, and tissue regeneration [122]. So, therapeutic release can be synchronized with local biological activity by incorporating enzyme-cleavable linkages into hydrogel networks. This hence improves treatment specificity and reduces premature cargo loss. For example, Zhang et al. developed an injectable, catechol-functionalized adhesive hydrogel incorporating MMP-responsive gelatin nanoparticles to be used for wound healing [123]. The gelatin nanocarriers remained stable under physiological conditions but underwent enzymatic degradation in MMP-rich wound environments. Therefore, curcumin was released on demand, while the dynamic adhesive hydrogel provided injectability, self-healing, and prolonged tissue retention. The hierarchical design of this material in particular illustrates how enzyme-responsive nanocarriers can be integrated with bio-inspired adhesive hydrogels to synchronize therapeutic release with tissue remodeling. So, this strategy enables hydrogels to adapt dynamically to tissue remodeling and maintain localized therapeutics.

Thermo-responsive systems have also been extensively developed for biomedical applications due to their ability to undergo reversible phase transitions near physiological temperatures [16]. Temperature-sensitive polymers can facilitate injectable delivery by maintaining low viscosity during administration followed by rapid gelation at body temperature [124,125]. For example, Liu et al. developed a hierarchical therapeutic platform by incorporating matrix metalloproteinase-9 (MMP-9)-responsive gelatin microspheres loaded with curcumin nanoparticles into a thermo-sensitive hydrogel for diabetic wound healing [126]. The thermo-responsive matrix in this material remained injectable at low temperature but rapidly gelled under physiological conditions. Such transition enabled sustained therapeutic release together with prolonged local retention at the wound site. Building upon this concept, Zhang et al. engineered an injectable thermo-sensitive catechol-functionalized hydrogel that underwent rapid in situ gelation upon exposure to physiological temperature [49]. Reversible sol-to-gel transition in this hydrogel facilitated syringe-based administration while ensuring conformal adaptation to irregular tissue defects and stable tissue adhesion. This is hence a clear demonstration of synergistic integration of thermo-responsive gelation and catechol-mediated bioadhesion for minimally invasive localized therapy. Collectively, these studies demonstrate that thermo-responsive hydrogels not only improve handling characteristics and post-injection localization but also provide an effective platform for sustained therapeutic delivery and prolonged tissue retention.

Overall, stimuli-responsive hydrogels represent a significant evolution beyond conventional adhesive biomaterials. These hydrogels actively sense and respond to biological signals rather than functioning solely as structural carriers, enabling adaptive therapeutic regulation within complex microenvironments. So, integration of catechol-mediated adhesion with environmentally responsive functionalities establishes a versatile platform for the development of next-generation smart biointerfaces capable of achieving more precise, effective, and personalized localized therapeutic interventions.

## 4. Therapeutic Applications

**Figure 4:**
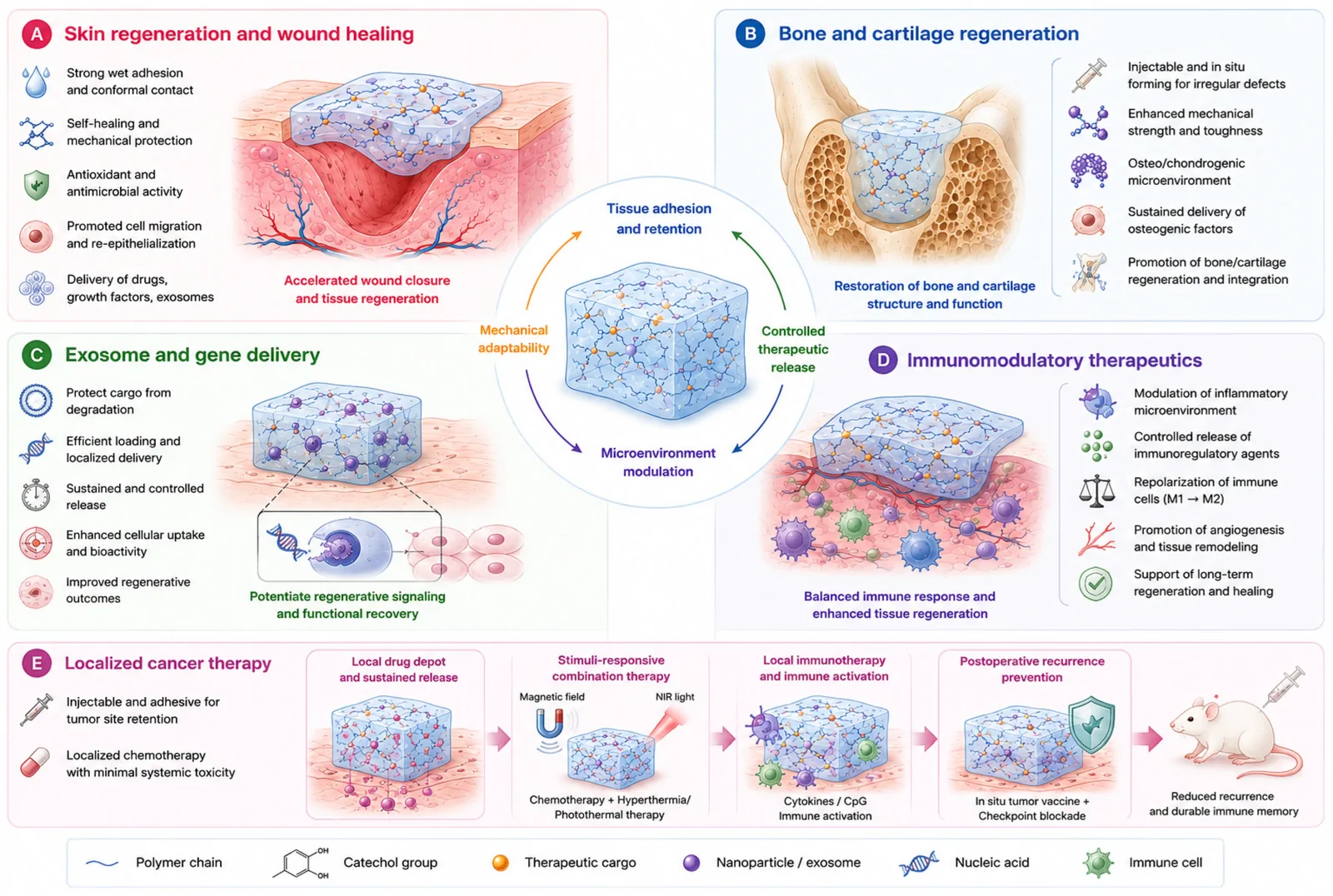
Therapeutic applications of bio-inspired adhesive hydrogels in regenerative medicine and localized therapy. Representative applications include (**A**) skin regeneration and wound healing, (**B**) bone and cartilage regeneration, (**C**) exosome and gene delivery, (**D**) immunomodulatory therapy, and (**E**) localized cancer therapy. These systems combine tissue adhesion and retention with controlled therapeutic delivery, mechanical adaptability, and microenvironment-responsive functions to support regenerative outcomes and localized treatment, including sustained chemotherapy, combination therapy, immune modulation, and postoperative tumor recurrence prevention.

Convergence of catechol-mediated adhesion, advanced hydrogel engineering, and stimuli-responsive functionalities has significantly expanded the therapeutic potential of bio-inspired adhesive hydrogels [49,81,127]. These systems have emerged as versatile platforms for addressing complex clinical challenges in regenerative medicine, as they provide robust tissue retention, adaptable mechanical properties, and programmable localized therapeutic delivery [128,129]. In particular, their ability to establish stable tissue interfaces while simultaneously serving as localized therapeutic depots has made broad applications in wound repair, musculoskeletal regeneration, emerging biologic therapies, and localized cancer treatment possible [81,130]. The following sections highlight representative therapeutic applications that demonstrate how recent advances in adhesive hydrogel design are translating into improved regenerative outcomes and next-generation localized treatment strategies (Figure 4).

### 4.1. Skin Regeneration and Wound Healing

Skin wound healing represents one of the most extensively investigated applications of bio-inspired adhesive hydrogels [49]. Successful wound management requires biomaterials capable of maintaining stable contact with moist tissue surfaces while simultaneously supporting tissue regeneration and localized therapeutic delivery. Conventional wound dressings, however, often exhibit insufficient adhesion and limited capacity to actively regulate the wound microenvironment. Hence, they exhibit restricted therapeutic efficacy [131]. These challenges have motivated the development of catechol-functionalized adhesive hydrogels that combine robust wet adhesion with multifunctional therapeutic capabilities.

Catechol-mediated adhesion enables hydrogel dressings to firmly attach to hydrated wound surfaces, minimizing material displacement and prolonging local retention of therapeutic agents [49]. At the same time, the hydrated polymer network provides an extracellular matrix-mimicking microenvironment supporting cell migration, proliferation, and re-epithelialization. This, in turn, facilitates tissue repair [5,8].

Recent advances in hydrogel engineering have further improved wound healing by integrating injectable, self-healing, nanocomposite, and stimuli-responsive functionalities to adhesive hydrogel platforms [42,49,81]. These design strategies enable hydrogels to conform to irregular wound geometries, dynamically respond to pathological microenvironments, and provide sustained delivery of antimicrobial agents, anti-inflammatory drugs, antioxidants, growth factors, and extracellular vesicles. For example, Duan et al. developed an injectable catechol-functionalized hyaluronic acid hydrogel capable of integrating tissue adhesion, self-healing, shape adaptability, and sustained therapeutic delivery. The authors, in particular, designed this hydrogel to comprehensively regulate the diabetic wound microenvironment [132]. Notably, this multifunctional hydrogel promoted fibroblast proliferation, reduced oxidative stress and inflammation, inhibited bacterial infection, enhanced angiogenesis, and accelerated wound healing. This is hence a demonstration of how multiple engineering strategies can be successfully combined within a single adhesive hydrogel platform.

Recent studies have demonstrated that these multifunctional hydrogels effectively modulate inflammation, reduce oxidative stress, promote angiogenesis, and accelerate collagen deposition and tissue remodeling. Hence, they significantly improve wound healing outcomes [81,119,132]. Recently, Zhou et al. developed a dual glucose/ROS-responsive injectable adhesive self-healing hydrogel that integrated photothermal antibacterial activity with microenvironment-responsive therapeutic regulation [119]. The hydrogel efficiently scavenged excessive ROS, promoted macrophage polarization toward a pro-regenerative M2 phenotype, enhanced angiogenesis and collagen deposition, and ultimately accelerated infected diabetic wound healing by orchestrating multiple stages of the regenerative process. As demonstrated here, bio-inspired adhesive hydrogels have evolved out of passive wound coverings into active therapeutic biointerfaces capable of orchestrating multiple stages of skin regeneration.

Meanwhile, adhesive hydrogels are increasingly being used beyond their usual application in conventional drug delivery, as carriers for advanced regenerative therapeutics, including growth factors, extracellular vesicles, and stem cell-derived bioactive factors [133,134,135]. The combination of prolonged tissue retention and controlled therapeutic release enhances local bioavailability and supports sustained regenerative signaling at wound sites. For example, Wang et al. developed an in situ photo-crosslinked catechol-functionalized GelMA hydrogel loaded with mesenchymal stem cell-derived extracellular vesicles (MSC-EVs) for diabetic wound healing [136]. This adhesive hydrogel enhanced angiogenesis, collagen deposition, and skin appendage regeneration and restored the wound microenvironment by improving EV retention and providing sustained local delivery. As a result, it significantly accelerated tissue repair compared to free EV administration. So, it is clear that bio-inspired adhesive hydrogels have evolved out of passive wound coverings into multifunctional therapeutic biointerfaces capable of actively promoting tissue repair and regeneration.

Overall, integration of robust tissue adhesion with programmable therapeutic functions has established catechol-based adhesive hydrogels as a versatile platform for advanced wound care and regenerative medicine. Continued advances in material engineering are expected to further expand their clinical potential for treating both acute and chronic skin wounds.

### 4.2. Bone and Cartilage Regeneration

Bone and cartilage regeneration remains a major challenge in medicine because successful repair requires coordinated structural support, localized therapeutic signaling, and long-term tissue remodeling [33,137]. Musculoskeletal defects are continuously exposed to mechanical loading and possess limited intrinsic regenerative capacity, unlike soft tissues. This requires biomaterials that can remain stably integrated while supporting sustained biological activity [138,139]. These requirements have stimulated development of bio-inspired adhesive hydrogels that combine robust tissue adhesion with regenerative functionality.

Catechol-functionalized hydrogels are particularly well suited for musculoskeletal applications because they establish strong adhesion to wet tissue surfaces and conform to irregular defect geometries. In effect, these hydrogels improve material retention and integration after implantation [49,81]. Their injectable nature further enables minimally invasive administration and in situ defect filling. Hence, they are attractive for treating complex bone and cartilage defects where conventional scaffolds often exhibit limited adaptability [58,140].

Recent advances have further enhanced the regenerative performance of adhesive hydrogels through incorporation of bioactive nanomaterials and therapeutic cargoes. Nanomaterials such as layered double hydroxides (LDHs), nanoclays, and silica nanoparticles not only reinforce hydrogel networks but also regulate degradation, therapeutic release, and cell–material interactions [98]. Very recently, Zou et al. developed a multifunctional silk fibroin hydrogel incorporating MgFe-layered double hydroxide (MgFe-LDH)-based metal–organic framework (MOF) composites. The LDH component in these composites simultaneously enhanced mechanical properties, delayed hydrogel degradation, and provided sustained release of bioactive Mg^2+^ and Fe^3+^ ions [141]. Consequently, the hydrogel promoted osteogenesis and angiogenesis while suppressing osteoclast differentiation through activation of the BMP2/SMAD/RUNX2 signaling pathway. These properties of the hydrogel resulted in significantly enhanced vascularized bone regeneration in multiple bone defect models. In parallel, osteogenic and chondrogenic therapeutics, including growth factors, small molecules, extracellular vesicles, and nucleic acids, have been incorporated into adhesive hydrogels to provide localized and sustained biological stimulation. This incorporation was originally planned to enhance therapeutic efficacy and reduce repeated administration [142,143,144]. For example, Zheng et al. developed a polyphenol-mediated electroactive hydrogel incorporating metal–polyphenol network-armored exosomes. The engineered exosomes in these hydrogels were protected from oxidative degradation and efficiently escaped lysosomal sequestration after cellular uptake [145]. This strategy and the sustained local delivery from the hydrogel matrix significantly enhanced osteogenic differentiation and bone regeneration by improving the stability and bioavailability of therapeutic exosomes within the inflammatory bone microenvironment.

So, emerging adhesive hydrogels actively regulate the regenerative microenvironment by promoting cell recruitment, directing osteogenic and chondrogenic differentiation, and coordinating extracellular matrix formation and tissue remodeling rather than acting solely as passive therapeutic delivery vehicles [12,146]. Integration of adhesive chemistry with multifunctional nanocomposite engineering has therefore transformed these materials into regenerative biointerfaces. These biointerfaces are capable of simultaneously providing structural support and biological instruction during musculoskeletal healing.

Overall, the combination of bio-inspired adhesion, injectable delivery, and multifunctional therapeutic regulation has established adhesive hydrogels as a promising platform for bone and cartilage regeneration. Continued advances in material design and bioactive integration are hence expected to further improve functional tissue restoration and accelerate clinical translation.

### 4.3. Emerging Regenerative Therapies

Rapid development of regenerative biologics has expanded the role of bio-inspired adhesive hydrogels beyond conventional drug delivery and tissue engineering [49,81]. Notably, adhesive hydrogels are increasingly being engineered as therapeutic biointerfaces capable of dynamically interacting with the local microenvironment and directing tissue regeneration, rather than functioning solely as localized delivery vehicles [5,147]. This emerging concept has accelerated the integration of advanced biologics, including extracellular vesicles, nucleic acid therapeutics, and immunomodulatory agents, into multifunctional adhesive hydrogel platforms.

Extracellular vesicles have attracted considerable attention compared to other emerging therapeutics mentioned above, owing to their ability to regulate intercellular communication without limitations associated with cell transplantation. Li et al. developed a peptide-modified adhesive hydrogel (Exo-pGel) for localized delivery of human mesenchymal stem cell-derived exosomes [148]. This adhesive hydrogel effectively immobilized exosomes within the hydrogel matrix, enabling prolonged local retention and sustained release of therapeutics. It also simultaneously mitigated inflammation and oxidative stress in the injured tissue microenvironment. As a result, the Exo-pGel platform significantly enhanced functional tissue regeneration compared to systemic exosome administration. This situation highlights potential use of adhesive hydrogels as effective carriers in cell-free regenerative therapies. Meanwhile, localized gene delivery using plasmid DNA, messenger RNA, small interfering RNA, and gene-editing systems has enabled sustained modulation of regenerative signaling pathways while minimizing systemic exposure [149]. Peng et al. developed an adhesive GelMA/snail mucus hydrogel incorporating engineered extracellular vesicles loaded with STAT1-siRNA for localized gene therapy [150]. The hydrogel served as a bioadhesive depot that enabled sustained release of targeted siRNA-loaded extracellular vesicles. It also enhanced tissue retention at the injury site. Local STAT1 gene silencing effectively suppressed M1 macrophage polarization and inflammatory signaling, promoting functional tissue regeneration. In essence, this study highlights how adhesive hydrogels can integrate localized gene delivery with microenvironmental modulation to improve regenerative outcomes. In parallel, immunomodulatory strategies that actively regulate macrophage polarization and inflammatory responses have further expanded the therapeutic capabilities of adhesive hydrogels beyond structural tissue repair. Zhou et al. developed a snail mucus-inspired adhesive AFG/GelMA hydrogel in which the adhesive mechanism was derived from natural snail glycosaminoglycan rather than conventional catechol chemistry [151]. The bioinspired hydrogel exhibited strong wet tissue adhesion through amide, hydrogen-bonding, and ionic interactions. It also simultaneously suppressed inflammatory cytokines and promoted M2 macrophage polarization. As a result, it markedly accelerated diabetic wound healing. This study demonstrates that emerging bio-inspired adhesive mechanisms beyond catechol chemistry can also serve as effective immunomodulatory platforms for tissue regeneration.

Collectively, these advances have transformed adhesive hydrogels from passive biomaterial scaffolds into multifunctional therapeutic biointerfaces capable of coordinating biological signaling, immune regulation, and tissue regeneration. Continued integration of emerging biologics with advanced hydrogel engineering is expected to accelerate development of precision regenerative therapies for increasingly complex clinical applications.

### 4.4. Localized Cancer Therapy

Localized cancer therapy represents an emerging application of bio-inspired adhesive hydrogels, particularly for improving drug retention at tumor sites and preventing postoperative recurrence. Compared with systemic administration, injectable adhesive hydrogels can establish localized therapeutic depots that prolong drug exposure while limiting systemic distribution. For example, a dopamine-functionalized injectable hydrogel based on oxidized carboxymethyl cellulose and carboxymethyl chitosan was developed for localized gemcitabine delivery in pancreatic cancer [152]. The hydrogel underwent rapid in situ gelation and provided sustained drug release for approximately one week, demonstrating the potential of catechol-containing injectable networks for maintaining therapeutically relevant drug concentrations within tumors.

Beyond conventional drug depots, catechol chemistry has enabled the development of multifunctional hydrogel systems that integrate localized chemotherapy with externally triggered therapeutic modalities. Dai et al. developed a magnetic nanocomposite hydrogel based on dopamine-conjugated hyaluronan and iron oxide nanoparticles, in which dynamic catechol–Fe(III) coordination simultaneously reinforced the hydrogel network and enabled magnetic-field-responsive doxorubicin release [72]. The resulting system combined sustained chemotherapy with magnetic hyperthermia, producing greater antitumor efficacy than either treatment alone. Similarly, polydopamine-containing adhesive hydrogels have been employed to integrate doxorubicin delivery with near-infrared photothermal therapy, enabling localized chemo-photothermal treatment while supporting postoperative wound repair [153]. These approaches illustrate how catechol-containing components can contribute not only to tissue retention but also to dynamic crosslinking, stimuli responsiveness, and multimodal therapeutic activity.

More recently, adhesive hydrogels have evolved toward localized immunomodulatory platforms designed to eliminate residual tumor cells and suppress postoperative recurrence [154]. A catechol-functionalized gelatin hydrogel incorporating doxorubicin and cytokine-releasing nanofibers promoted immune infiltration and enhanced immunochemotherapy in an osteosarcoma model. In another approach, a bioadhesive hydrogel containing dopamine-derived carbon quantum dot-supported Pd single-atom nanozymes and CpG oligonucleotides enabled localized catalytic immunotherapy and enhanced systemic antitumor immunity when combined with immune checkpoint blockade [155]. More recently, a catechol-containing, metal–phenolic microcapsule-reinforced hydrogel was designed as a postoperative in situ tumor vaccine, enabling pH-responsive doxorubicin release, immunogenic cell death, tumor-antigen capture, and dendritic-cell activation [155]. Combined with anti-PD-1 therapy, this platform suppressed postoperative recurrence and induced durable antitumor immune memory in a breast cancer model.

## 5. Future Perspectives and Conclusions

Several challenges remain in fully realizing bio-inspired adhesive hydrogels before their widespread clinical translation, despite remarkable advances in their study. Catechol-mediated adhesion provides robust wet adhesion under hydrated conditions. However, maintaining long-term functionality in dynamic physiological environments remains difficult because of continuous mechanical loading, tissue remodeling, and material degradation [49,81]. In addition, practical manufacturing and storage issues remain important translational barriers [55]. Catechol groups are susceptible to oxidation, raising potential concerns regarding their chemical stability during processing, sterilization, and long-term storage; such changes may affect crosslinking behavior and adhesive performance [55,156]. Scalable synthesis of dopamine-functionalized polymers also requires consistent control over the degree of substitution and batch-to-batch reproducibility [147]. These challenges become more complex for multicomponent systems incorporating nanomaterials or biologics, where sterilization compatibility, shelf-life stability, quality control, and regulatory evaluation of individual components and the final combination product must all be considered [147,156]. Addressing these translational barriers through standardized manufacturing processes and regulatory frameworks will be essential for the successful commercialization of next-generation adhesive hydrogel platforms.

Looking forward, the field is expected to evolve beyond conventional mussel-inspired catechol chemistry to a broader spectrum of bio-inspired adhesive mechanisms. Emerging adhesive strategies inspired by natural systems, such as snail mucus, sandcastle worms, barnacles, and geckos, together with synthetic biomimetic chemistries, may be promising. These strategies provide improved tissue specificity, enhanced long-term adhesion, and expanded applicability across diverse biomedical environments [151,157,158]. Such developments are anticipated to further diversify the molecular toolbox available for the engineering of next-generation adhesive biomaterials.

Future adhesive hydrogels are also expected to transition from passive delivery systems into intelligent therapeutic biointerfaces capable of dynamically sensing and responding to changes in local microenvironment. Next-generation platforms may integrate biomarker-responsive, enzyme-responsive, and inflammation-responsive mechanisms to achieve feedback-controlled therapeutic regulation rather than simply providing sustained therapeutic release [5,74,114,146]. Combination of adaptive material behavior and localized delivery is expected to enable increasingly precise modulation of tissue repair and regeneration.

At the same time, advances in artificial intelligence (AI)-assisted materials discovery and precision regenerative medicine are likely to transform hydrogel design strategies. Machine learning-guided optimization, inverse materials design, and high-throughput computational screening may accelerate identification of hydrogel formulations with tailored physicochemical and biological properties [159,160,161]. These approaches will facilitate development of personalized adhesive hydrogel platforms capable of delivering individualized regenerative therapies with improved efficacy and safety when coupled with patient-specific biomarkers and disease-associated microenvironmental information.

In conclusion, bio-inspired adhesive hydrogels have rapidly evolved from simple tissue adhesives into multifunctional therapeutic biointerfaces. They are hence capable of integrating structural support, localized therapeutic delivery, and dynamic regulation of tissue regeneration. Continued advances in bio-inspired adhesion mechanisms, intelligent biomaterials engineering, and clinically translatable manufacturing strategies will further accelerate the application of these hydrogels as next-generation platforms for precision regenerative medicine.

## Figures and Tables

**Table 1:** Key catechol interactions in aqueous environments.

Interaction	Molecular Basis	Representative Partners	Biological Significance
Hydrogen bonding	Catechol hydroxyl groups act as hydrogen bond donors and acceptors.	Hydroxyl (–OH), Carboxylate (–COO^−^), Phosphate (–PO_4_^2−^), and Amine (–NH_2_/–NH_3_^+^).	Promotes wet adhesion through reversible intermolecular interactions.
Metal coordination	Bidentate coordination through adjacent catechol oxygens.	Fe^3+^, Ti^4+^, and Al^3+^ (also Ca^2+^, Mg^2+^, and Zn^2+^ under specific conditions).	Dynamic crosslinking, enhanced mechanical strength, self-healing.
π–π stacking & hydrophobic interactions	Aromatic interactions between catechol rings and aromatic surfaces.	Aromatic amino acids (Phe, Tyr, and Trp), aromatic polymers, and graphitic materials	Improves interfacial stabilization and cohesive interactions.
Oxidation to quinone	Catechol is oxidized into reactive o-quinone species.	Dissolved oxygen, ROS, and oxidants	Enables subsequent covalent coupling reactions.
Covalent coupling	Michael addition and Schiff-base reactions with quinones.	Amines, thiols, and imidazole-containing residues	Forms stable covalent bonds for durable adhesion and network stabilization.

Abbreviations: Phe, phenylalanine; Tyr, tyrosine; Trp, tryptophan; ROS, reactive oxygen species.

**Table 2:** Representative catechol-functionalized hydrogel networks for localized therapeutic delivery.

Polymer Backbone	Intrinsic Biological Characteristics	Major Catechol-Mediated Advantages	Typical Crosslinking Strategies	Representative Therapeutic Applications
Hyaluronic acid (HA)	ECM-mimetic, CD44 binding, and highly hydrophilic	Strong tissue adhesion, prolonged retention, and injectable network formation	Oxidative coupling, Schiff-base, and photo-crosslinking	Bone regeneration, cartilage repair, wound healing, and exosome delivery
Chitosan	Mucoadhesive, antimicrobial, and hemostatic	Enhanced wet adhesion and tissue sealing	Oxidative crosslinking and dynamic covalent bonding	Wound dressings, mucosal delivery, and hemostatic materials
Alginate	Mild gelation, biocompatibility, and ion responsiveness	Improved interfacial adhesion and local retention	Ionic crosslinking and metal-catechol coordination	Drug delivery, tissue repair, and cell encapsulation
Gelatin	Cell-interactive motifs (RGD) and biodegradability	Enhanced biointegration and tissue attachment	Enzymatic, photo-crosslinking, and catechol oxidation	Tissue engineering and regenerative medicine
PEG	Synthetic, highly tunable, and reproducible	Controllable adhesion and mechanical properties	Thiol-catechol coupling and photo-crosslinking	Surgical sealants and localized drug delivery
Composite systems	Multifunctionality through hybrid design	Simultaneous adhesion, reinforcement, and controlled release	Dual-network and nanocomposite crosslinking	Precision therapeutics and smart biointerfaces

Abbreviations: ECM, extracellular matrix; CD44, cluster of differentiation 44; HA, hyaluronic acid; RGD, arginine–glycine–aspartic acid; PEG, polyethylene glycol.

**Table 3:** Critical design parameters governing adhesive performance of catechol-functionalized hydrogels.

Design Parameter	Engineering Role	Benefits of Increasing Parameter	Potential Limitations	Design Considerations for Localized Therapeutic Delivery
Catechol density	Provides adhesive functionalities	Stronger tissue adhesion and cohesive interactions	Excessive oxidation, reduced chain mobility, and brittleness	Optimize adhesion without compromising injectability and flexibility
Catechol oxidation state	Regulates catechol–quinone balance	Enhanced covalent tissue bonding and network stabilization	Overoxidation may reduce reversible adhesion and biocompatibility	Control redox environment and oxidation kinetics
Crosslinking density	Governs network architecture	Improved mechanical integrity and retention	Reduced conformability and therapeutic diffusion	Balance structural stability and mass transport
Mechanical properties	Determines resistance to physiological stress	Enhanced durability and tissue fixation	Excessive stiffness may impair tissue integration	Match mechanical properties to target tissue
Degradation behavior	Controls material persistence	Prolonged therapeutic retention	Delayed remodeling and clearance	Synchronize degradation with tissue healing processes

**Table 4:** Quantitative comparison of representative catechol-based adhesive hydrogel systems, including their network chemistry, adhesive and mechanical properties, gelation behavior, degradation/stability, and therapeutic loading and release characteristics.

Hydrogel System	Polymer/Catechol Chemistry	Crosslinking Mechanism	Adhesion Test/Strength	*G*′	Gelation Time	Degradation	Therapeutic Loading/Release	Ref.
Dopamine-conjugated dialdehyde hyaluronic acid hydrogel	Dialdehyde HA conjugated with dopamine; catechol DS up to 45%	NaIO_4_-mediated oxidative catechol crosslinking	Lap shear, wet porcine skin; 90.0 ± 6.7 kPa	NR	<60 s	≤31.1% weight loss at 29 d in hyaluronidase	NR	Zhou et al., 2020, [60]
Catechol-modified methacrylated hyaluronic acid hydrogel	Methacrylated HA conjugated with dopamine; dopamine substitution 46.7%	Blue-light-induced thiol–ene crosslinking with catechol-associated interactions	Lap shear; 2.9 MPa on PET; porcine skin also tested	NR	32 s	NR	NR	Yang et al., 2024, [61]
Tannic acid-reinforced catechol-modified hyaluronic acid hydrogel	HA–catechol conjugate; catechol DS 9.6% + tannic acid	NaIO_4_-mediated oxidative crosslinking + TA-mediated interactions	Adhesion test, porcine skin; ~10 kPa	*G*′ = 881 Pa	NR	Almost complete degradation within 3 d in 50 U/mL hyaluronidase	NR	Gwak et al., 2021, [62]
Dopamine-grafted gelatin/graphene oxide nanocomposite hydrogel	Dopamine-grafted gelatin + graphene oxide	H_2_O_2_/HRP-mediated oxidative catechol coupling + reversible catechol–boronate interactions	Lap shear, hog skin; 16.2 ± 2.4 kPa	*G*′ = 2319 Pa	NR	NR	NR	Han et al., 2021, [63]
Visible-light-crosslinked caffeic acid-modified gelatin hydrogel	Gelatin covalently modified with caffeic acid	Eosin Y/triethanolamine/N-vinylcaprolactam-mediated visible-light crosslinking + secondary catechol coupling	Lap shear, porcine skin; 18.33 ± 0.42 kPa	NR	2 min irradiation	Enhanced enzymatic stability; quantitative value NR	NR	Lin et al., 2025, [44]
Caffeic acid oligomer-conjugated gelatin hydrogel	Gelatin conjugated with caffeic acid oligomers	Thermosensitive physical gelation followed by NaIO_4_-mediated oxidative crosslinking	Pig tissue adhesion; ~3-fold higher than GelMA	NR	Rapid thermosensitive gelation; exact value NR	<3% polymer release after oxidative crosslinking in aqueous stability test	NR	Montazerian et al., 2023, [64]
Gallic acid-grafted chitosan self-crosslinked hydrogel	Chitosan grafted with gallic acid; GA content up to 172.6 ± 11.1 mg/g	Oxygen-induced polyphenol oxidation followed by Schiff-base/Michael-addition crosslinking	Lap shear, porcine skin; 84.65 ± 1.67 kPa	Mechanical moduli >100 Pa	~3 h under air exposure	NR	NR	Sun et al., 2022, [65]
Catechol-modified/oxidized chitosan double-crosslinked hydrogel	Catechol-modified chitosan + oxidized chitosan; catechol substitution 4.6–60.7%	Catechol–Fe^3+^ coordination + Schiff-base crosslinking	Lap shear, porcine tissue; 45.6 kPa	NR	NR	NR	NR	Zhao et al., 2025, [66]
Hydrocaffeic acid-modified chitosan/polyethylene glycol double-network hydrogel	Hydrocaffeic acid-conjugated chitosan; catechol grafting 15% + 4-arm PEG	NaIO_4_-mediated catechol oxidation + strain-promoted azide–alkyne cycloaddition	Lap shear, porcine skin; 7.32 ± 1.0 kPa	*G*′ = 410 Pa	30 s	51.6 ± 5.7% remaining at 21 d	NR	Li et al., 2022, [67]
Catechol-functionalized poly(ethylene glycol) oxime hydrogel	Aldehyde-, aminooxy-, and dopamine-derived catechol-functionalized 8-arm PEG	Rapid oxime crosslinking + secondary catechol-mediated interactions	Ex vivo porcine cardiac tissue retention; quantitative strength NR	*G*′ = 8.1 ± 1.0 kPa	<3 s	0.8 ± 3.4 wt% mass loss at 28 d in PBS	NR	Fujita et al., 2021, [68]
TGF-β3-loaded catechol-modified quaternized chitosan/4-arm poly(ethylene glycol) hydrogel	Catechol-modified quaternized chitosan + benzaldehyde-terminated 4-arm PEG; TGF-β3 1 ng/mL	Dynamic Schiff-base crosslinking	90° peel, glass; 28.5 ± 2.1 J/m^2^	Young’s modulus = 10.2 ± 0.8 kPa; tensile strength = 15.3 ± 1.2 kPa	2–3 min	28.4 ± 2.1% at 7 d; 55.7 ± 1.8% at 14 d; 82.3 ± 2.5% at 21 d	TGF-β3 release: 58.4 ± 2.7% at 3 d; 78.2 ± 2.9% at 7 d; 91.5 ± 3.3% at 14 d	Yang et al., 2025, [69]
Polydopamine–silicate/cellulose nanocrystal-reinforced polyacrylamide nanocomposite hydrogel	Polyacrylamide containing polydopamine-intercalated silicate nanoflakes, cellulose nanocrystals, and calcium sulphoaluminate	Radical polymerization + CSA-derived nanocrystal-mediated ionic/H-bond crosslinking + catechol-mediated tissue interactions	Underwater lap shear, porcine skin; 30.7 ± 3.8 kPa	*G*′ = 78.96 kPa for optimal CSA/AM = 3	<5 min	~34.3% degradation at 15 d for CSA/AM = 3	Curcumin-loaded nanofiber composite: 2.0% release at 0.5 h; sustained release to 120 h	Chen et al., 2021, [70]
Dopamine-grafted phosphorylated nanocellulose/quaternized chitosan nanocomposite hydrogel	Poly(acrylic acid-co-acrylamide) network containing dopamine-grafted phosphorylated cellulose nanofibers and quaternized chitosan	Covalent polymer network + phosphate–cation electrostatic interactions + hydrogen bonding/catechol-mediated interfacial adhesion	Wet lap shear, porcine skin; 78.82 ± 2.55 kPa	Tensile strength = 118.75 kPa; compressive strength = 730.63 kPa at highest DA-PCNF content	40 min thermal crosslinking at 80 °C	NR	No therapeutic cargo; intrinsic antibacterial/antioxidant functionality	Liu et al., 2026, [71]
Doxorubicin-loaded dopamine-modified hyaluronan/iron oxide magnetic nanoparticle nanocomposite hydrogel	Dopamine-conjugated hyaluronan + Fe(III)-containing iron oxide magnetic nanoparticles; DOX 0.2 mg/mL	Dynamic DOPA–Fe(III) coordination between HA-DOPA and magnetic nanoparticles + secondary noncovalent DOX interactions	Tissue adhesion strength NR; prolonged in vivo retention reported	*G*′ ≈ 160 Pa	Gel formation observed within 24 h	NR	DOX release: 10.3% at 1 d and 17.8% at 7 d without AMF; AMF accelerated release on demand	Dai et al., 2021, [72]

NR, not reported; DS, degree of substitution; HA, hyaluronic acid; PET, polyethylene terephthalate; TA, tannic acid; HRP, horseradish peroxidase; GelMA, gelatin methacryloyl; GA, gallic acid; PEG, poly(ethylene glycol); PBS, phosphate-buffered saline; TGF-β3, transforming growth factor beta 3; CSA, calcium sulphoaluminate; AM, acrylamide; AMF, alternating magnetic field; DOX, doxorubicin; *G*′, storage modulus. Quantitative values are presented as reported in the original studies. Direct numerical comparison should be interpreted cautiously because adhesion substrates, testing methods, hydrogel compositions, and experimental conditions differ among studies. Mechanical properties are reported using the representative parameter provided in each original study.

**Table 5:** Design strategies for next-generation bio-inspired adhesive hydrogels.

Design Strategy	Engineering Rationale	Representative Approaches	Functional Advantages	Impact on Localized Therapeutic Delivery
Injectable systems	Enable minimally invasive administration and conformal filling of irregular defects	Shear-thinning hydrogels, injectable precursors, and catheter-deliverable systems	Improved handling, defect adaptation, and enhanced retention	Precise local administration and reduced surgical burden
In situ gelation	Form hydrogels directly at the target site after administration	Catechol oxidation, Schiff-base reaction, enzymatic crosslinking, and metal coordination	Enhanced tissue retention and localized depot formation	Sustained therapeutic localization
Dynamic covalent networks	Introduce reversible network remodeling according to physiological conditions	Schiff-base bonds, boronate esters, and reversible covalent chemistry	Stress dissipation, adaptability, and prolonged functionality	Improved durability in dynamic tissue environments
Self-healing mechanisms	Restore structural integrity following mechanical damage	Dynamic covalent bonding, host–guest interactions, and metal-catechol coordination	Autonomous repair and long-term stability	Extended therapeutic retention and performance
Nanocomposite reinforcement	Improve mechanical properties and multifunctionality	LDH, nanoclay, silica nanoparticles, liposomes, and polymeric nanoparticles	Mechanical reinforcement, bioactivity, and cargo protection	Enhanced therapeutic efficacy and sustained release
Stimuli-responsive functionalities	Enable environmental adaptation and on-demand therapeutic action	pH-, ROS-, enzyme-, and thermo-responsive systems	Triggered release and spatiotemporal control	Precision therapeutic delivery
Therapeutic cargo integration	Expand biological functionality beyond structural support	Small molecules, proteins, exosomes, and nucleic acids	Multifaceted therapeutic effects	Regenerative and disease-specific treatment

Abbreviations: LDH, layered double hydroxide; ROS, reactive oxygen species.

## Data Availability

No new data were created or analyzed in this study. Data sharing is not applicable to this article.
